# Picolinate-mediated immunomodulation: insights from Mendelian randomization on the role of NK cell percentage in the pathogenesis of lichen planus

**DOI:** 10.3389/fimmu.2024.1464479

**Published:** 2024-12-12

**Authors:** Jianye Cao, Tiantao Du, Jian Li, Baiyu Chen, Xianting Xie, Guoshu Zhang, Jia Feng, Tao Xu

**Affiliations:** ^1^ Clinical Medical College, Southwest Medical University, Luzhou, China; ^2^ Department of Thoracic Surgery, Hejiang County People’s Hospital, Luzhou, Sichuan, China; ^3^ Department of Laboratory Medicine, The Affiliated Hospital of Southwest Medical University, Sichuan Province Engineering Technology Research Center of Molecular Diagnosis of Clinical Diseases, Molecular Diagnosis of Clinical Diseases Key Laboratory of Luzhou, Luzhou, Sichuan, China; ^4^ Department of Thoracic Surgery, Affiliated Hospital of Southwest Medical University, Luzhou, China

**Keywords:** immune cells, Lichen Planus, mediation analysis, Mendelian randomization, plasma metabolites

## Abstract

**Background:**

Lichen planus (LP), an autoimmune disorder, remains incompletely understood in terms of its etiological mechanisms. This study aims to elucidate causal relationships among immune cell populations, plasma metabolites, and lichen planus using Mendelian randomization (MR) techniques.

**Methods:**

Employing a two-sample, two-step MR approach, with single nucleotide polymorphisms (SNP) serving as genetic instruments for both exposures and mediators, this study minimizes biases from confounding and reverse causality. Leveraging summary statistics from genome-wide association studies (GWAS) involving 731 immune cell traits (N = 3757), 1091 plasma metabolite traits (N = 8299), and lichen planus (N = 367668), inverse variance weighting (IVW) is adopted as the primary MR analytical method. The total effect of immune cells traits on LP is decomposed into direct and indirect effects mediated by plasma metabolites.

**Results:**

MR analysis reveals causal associations for 28 immune cell traits and 38 plasma metabolites with LP (*P_IVW_* < 0.05). Specifically, NK % lymphocyte shows a negatively correlated causal effect with LP (OR_IVW_ = 0.952; 95% CI: [0.910, 0.995], *P_IVW_* = 0.030). Among mediators, Picolinate significantly contributes, explaining 16.4% (95% CI: [28.3%, 4.54%]) of the association between NK % lymphocyte and LP.

**Conclusion:**

These findings support a potential protective causal effect of NK % lymphocyte on LP, partially mediated by Picolinate levels. Thus, interventions targeting Picolinate levels may mitigate LP burden attributed to low NK % lymphocyte counts. This study provides new evidence and insights into the pathogenesis of lichen planus, advancing our understanding of its underlying mechanisms.

## Introduction

Lichen planus (LP) is an idiopathic inflammatory skin disease of unknown etiology with unique disease characteristics. It is characterized by purplish-red flat papules on the skin, mucous membranes and external genitalia, often associated with itching and pain, which in severe cases can lead to skin breakdown, ulceration and scarring (1–3). The course of LP is usually characterized by chronicity and recurrence, which has a serious impact on the quality of life and psychological well-being of patients (4). Currently, there is a lack of specific diagnostic tools and effective therapeutic options for LP. Therefore, further investigation of the pathogenesis of lichen planus and searching for potential therapeutic targets are of great significance in improving the diagnostic accuracy and therapeutic efficacy of LP.

From an immunological perspective, LP is generally recognized as an autoimmune disease mediated by T cells, in which inflammatory cells such as T helper cells, T cytotoxic lymphocytes, natural killer cells and dendritic cells play a central role (1). Mana et al. showed that CXCR3 and CCR5-mediated signaling pathways initiated by T cells and keratinocytes promote LP pathogenesis (5). Of course, this association may be mediated by multiple risk factors, such as metabolite levels. Nevertheless, the metabolite level-mediated contribution of immune cells to the pathogenesis of LP has not been reported. A recent study by Li et al. suggests that certain plasma metabolite levels may influence the development of LP, and metabolites uridine, taurine, glutamate, citric acid and LysoPC (18:1) may suggest a malignant transformation of oral lichen planus (6). This was also demonstrated in wang et al. (7). These metabolites may be involved in immune cell regulatory activity and function, thereby affecting the condition and severity of LP. For instance, taurine has been shown to be closely associated with the immune system (8, 9). Understanding the mediating mechanisms in the association of immune cells with LP will provide important information for LP treatment, for example, by synergistically intervening with immune cells and intermediary targets to reduce the risk of LP due to immune cells. Our currently available knowledge of mediated pathways is largely based on traditional observational studies that are susceptible to confounding and reverse causation, making it difficult to establish causality. Therefore, it remains uncertain to what extent the associations between immune cells and LP and its intermediate variables are subject to confounding or reverse causation.

Mendelian randomization (MR) is a novel analytical method for epidemiological studies that uses single nucleotide polymorphisms (SNP) identified in genome-wide association studies (GWAS) that are strongly correlated with exposure levels as instrumental variables (IV) in MR analyses to assess causation (10, 11). MR-derived estimates of the relationship between exposure and outcome are unlikely to be biased by unobserved confounding provided a range of assumptions are met (12). Recent studies have used MR analysis to confirm a causal relationship between gut flora, schizophrenia, etc. and LP (13, 14).

In this study, we used MR technique to first select SNP associated with specific exposures as instrumental variables. This was followed by two-sample MR analyses to reveal causal links between immune cells, plasma metabolites, and LP. To deeply understand LP etiology, particularly the role of metabolites in mediating immune-metabolic interactions in LP pathogenesis, we used a two-step MR approach. This method systematically assesses the mediating effects of candidate metabolites in the causal chain between immune cells and LP and constructs a refined etiological network. In summary, this study aims to analyze the complex causal relationship between LP and related biomarkers using cutting-edge genetic epidemiological methods. It focuses on the pathological process of the synergistic interaction between immune regulation and metabolic disorders. The expected results will deepen the understanding of the direct etiology and indirect pathways of LP, improve the precision of the etiological evidence, and support the development of targeted prevention and treatment strategies, as well as the optimization of personalized medicine.

## Methods

### Study design

**Figure 1:**
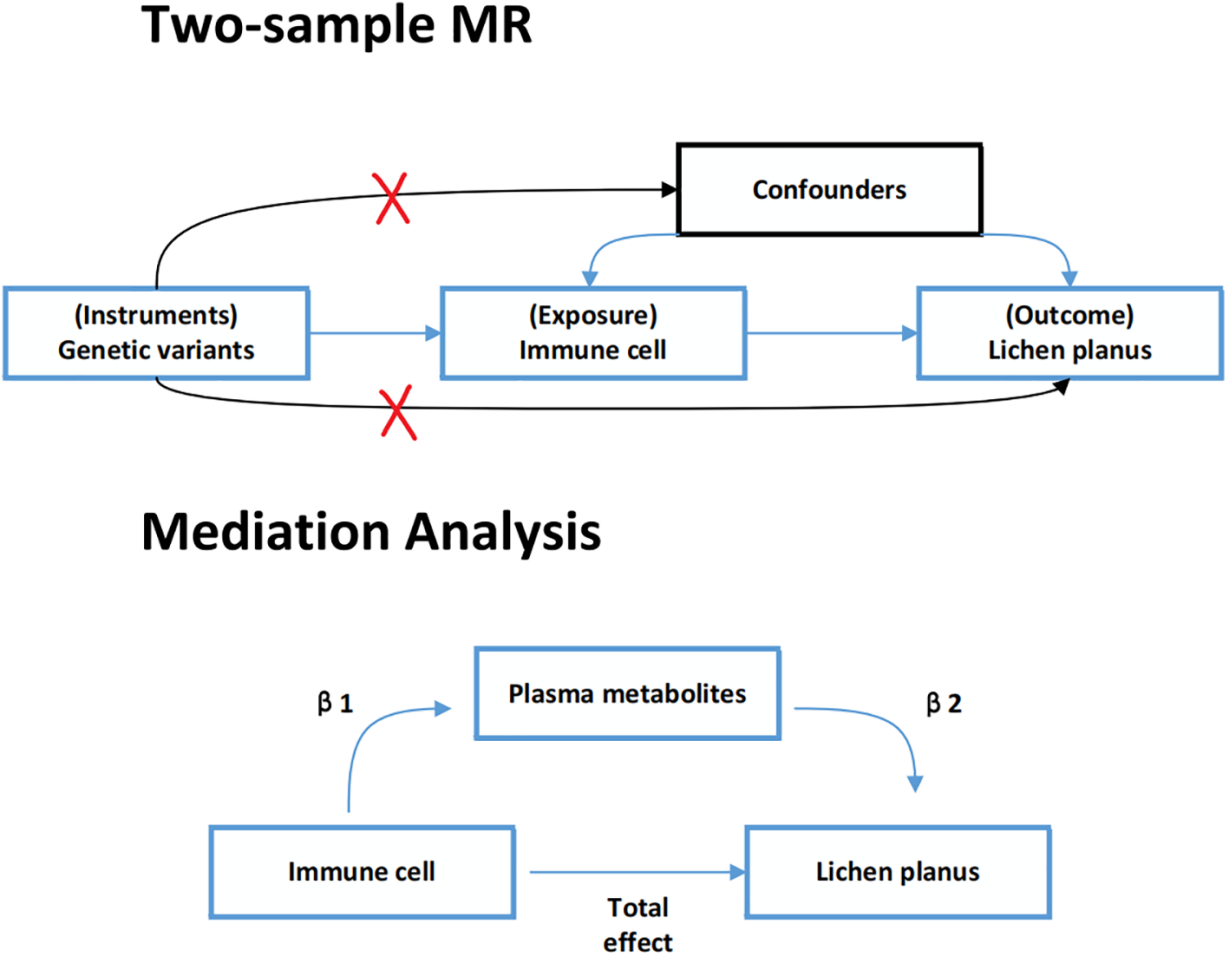
Assumptions and design of two-sample and mediated Mendelian randomization (MR) analyses.

In this study, all data were sourced from publicly accessible GWAS summary data. We extracted SNPs associated with immune cell traits and plasma metabolites to use as IV. Firstly, two-sample MR analyses were taken to explore the causal relationship between immune cell traits and lichen planus. Secondly, a two-step MR analysis was used to determine whether plasma metabolites could mediate this causal relationship. The detailed plan is shown in **Figure 1**. For the IV used in the MR analysis three main assumptions had to be satisfied (15, 16): (1) relevance: IV was strongly associated with exposure; (2) exclusivity: it was associated with outcome only through exposure; (3) independence: IV was not associated with any confounding factors. Additionally, each publicly available GWAS used in this MR study received ethical approval from the relevant authorities. The STROBE-MR (Strengthening the Reporting of Observational Studies in Epidemiology using Mendelian Randomization) checklist was completed for this observational study (**Supplementary Table S1**) (12).

### GWAS summary data sources

The summary statistics for immune cell characteristics were obtained from the GWAS Catalog (https://gwas.mrcieu.ac.uk/). The data were derived from 3757 Sardinians, a Mediterranean population with specific genetic traits that may influence immune system characteristics. The participants included both males and females, with an age range spanning from young adults to elderly individuals. Key demographic characteristics such as age, sex distribution, and body mass index (BMI) were recorded for all individuals, and additional factors like smoking status, physical activity, and health conditions (e.g., autoimmune diseases or chronic infections) were also considered as potential covariates in the analysis. The comprehensive study covered 731 immune cell characteristics across seven panels: Mature B cell, cDC, Maturation stages of T cell, Monocyte, Myeloid cell, TBNK, and Treg. It also included four types of immunological characteristics: median fluorescence intensity (MFI = 389), relative cellularity (RC = 192), absolute cellularity (AC = 118), and morphological parameters (MP = 32) (17).

The summary statistics for plasma metabolites were obtained from the GWAS Catalog (https://www.ebi.ac.uk/gwas/), including 1091 plasma metabolites for 8299 European subjects (18). The cohort consisted of individuals from diverse European populations, with age and sex distributions carefully recorded. The study included both healthy individuals and those with underlying conditions, such as metabolic disorders or cardiovascular disease, to explore the impact of disease status on metabolic profiles. Demographic variables, including age, sex, ethnicity, smoking, alcohol consumption, diet, and physical activity, were carefully controlled for, ensuring the robustness of the findings. This dataset also considered medication usage and potential confounders like the participants’ clinical history (e.g., previous diagnosis of obesity or diabetes). The plasma metabolites were categorized into molecules (N = 21) and unknown molecules (N = 220), classification into lipid (N = 395), amino acid (N = 210), xenobiotics (N = 130), nucleotides (N = 33), cofactors and vitamins (N = 31), carbohydrates (N = 22), peptides (N = 21), and energy (N = 8) fractions. The diversity in the population and the detailed demographic characteristics enhance the generalizability of the study’s findings.

The GWAS summary statistics related to lichen planus were obtained from FinnGen GWAS (https://r9.finngen.fi/) under the identifier finngen_R9_L12_LICHENPLANUS. In the FinnGen GWAS database, the diagnosis of lichen planus is typically based on ICD-10. The diagnosis of lichen planus was determined by the ICD-10 code L43 in the medical records. The dataset includes a sample of 3597 patients with lichen planus from the European population and 364071 controls. This dataset also includes demographic information such as age, sex, and ethnicity. Lifestyle factors, such as smoking, alcohol use, and physical activity, were also recorded for participants in both the case and control groups, as these factors can influence the clinical presentation of lichen planus. These data underwent rigorous quality control to ensure the reliability of the study results.

### Instrumental variable selection

To ensure the stability and accuracy of the results, SNPs were quality-checked to obtain compliant IV. Genome-wide significance threshold-associated SNP (*P* < 5e-08) were selected as candidate IV. Unfortunately, the number of compliant IV was very low. Therefore, we set the filtering condition for immune cell characterization of SNP as IV to *P* < 1e-05 (19), and SNP in plasma metabolites to *P* < 1e-05 (20). More stringent *p-values* (*P* < 5e-08) were used to determine the IV for lichen planus. To avoid bias due to linkage disequilibrium (LD), we used a clumping method with r2 = 0.001 and a window size of 10000 kb to obtain independent SNP (21). The strength of the selected SNP was evaluated using the F-statistic, and SNP with an F value of less than 10 were removed to avoid the effects of weak instrumental variables on the MR analysis. The F-statistic formula used was F = [R^2^ × (n-k-1)]/[k × (1-R^2^)]. In this formula, R^2^ represents the proportion of variability explained by each SNP, n represents the size of the GWAS sample, and k represents the number of SNP (22).

### Two-sample Mendelian randomization

MR methods were used to evaluate the causal relationships between immune cells, plasma metabolites, and lichen planus. For exposures containing multiple IV, causality was inferred using inverse variance-weighted (IVW) (23), Simple mode (24), MR-Egger (25), weighted median (26),and weighted mode (26) methods. IVW is considered to have the highest statistical efficacy (27). Therefore, the most accurate IVW method was used as the first choice (*P* < 0.05 was considered a significant association between exposure and outcome), and the remaining four methods were used as supplements. Meanwhile, MR-Egger also tests for pleiotropy. The intercept provides an estimate of the average pleiotropy of genetic variants (25). Additionally, the weighted median method can provide a valid causal estimate even if up to 50% of the IV are invalid (26). This study selects results with *P_IVW_ <*0.05, where the other four methods are consistent with the IVW method, as potentially indicative of a significant causal association.

Sensitivity analysis was also performed to assess the stability of causal relationships. Horizontal pleiotropy was assessed using MR-Egger regression and MR-PRESSO (28). A non-zero intercept in the MR-Egger regression indicates directional pleiotropy (25). Cochran’s Q test was used to assess heterogeneity among IV. Additionally, leave-one-out sensitivity analyses were performed to assess whether individual SNP influenced the causal analysis (23). Ultimately, the results obtained from the MR analysis must also satisfy the sensitivity analysis.

Furthermore, to account for multiple testing in the IVW analysis, we applied the Bonferroni correction to adjust the significance threshold and control for type I errors (29). The adjusted significance level(Q) was calculated as: Q=0.05/N. where N represents the total number of independent causal tests performed. The *P* from the IVW analysis were compared against this adjusted threshold to determine statistical significance. Results with *P* below Q were considered statistically significant after correction, while results below the conventional threshold (*P* =0.05) but above Q were interpreted with caution. This study used R language version 4.3.1.

### Reverse Mendelian randomization analysis

Reverse Mendelian randomization analysis was performed to investigate whether flat moss has a causal effect on identified immune cells. SNP associated with flat moss were screened as exposure IV, and identified immune cells were considered as endpoints. The reverse MR analysis followed a similar procedure to the previously described MR analysis.

### Mediation analysis

Mediation analyses, which assess the pathway from exposure to outcome through a mediator, can help explore potential mechanisms underlying the effect of exposure on outcome (30). This study focused on plasma metabolites as mediators to explore whether they mediate a causal pathway from immune cells to LP. The causal relationships between immune cells and plasma metabolites were assessed using two-sample MR analysis to obtain β1, followed by an assessment of causal relationships between plasma metabolites and LP to obtain β2. The mediator effect was then calculated using a two-step MR: mediator effect = β1 × β2. The total effect was determined when assessing the causal relationship between exposure and outcome, and the direct effect was calculated as: direct effect = total effect - mediator effect. The mediated proportion was calculated using the formula: mediated proportion = (mediator effect/total effect) × 100%. The 95% confidence intervals (CI) for the mediating effect and the mediated proportion were estimated using the delta method (30).

## Results

### Selection of instrumental variables

According to the selection criteria for IV, we removed palindromic variants. After clumping and harmonization, we identified a total of 16959 SNP (immune cell signature, *P*<1e-05) and 27534 SNP (plasma metabolite signature, *P*<1e-05) as IV. These IV consistently had F-statistics greater than 10, and the number of SNP with immune cell signatures and plasma metabolite signatures was greater than or equal to three.

### Total effect of immune cell traits on lichen planus

**Figure 2:**
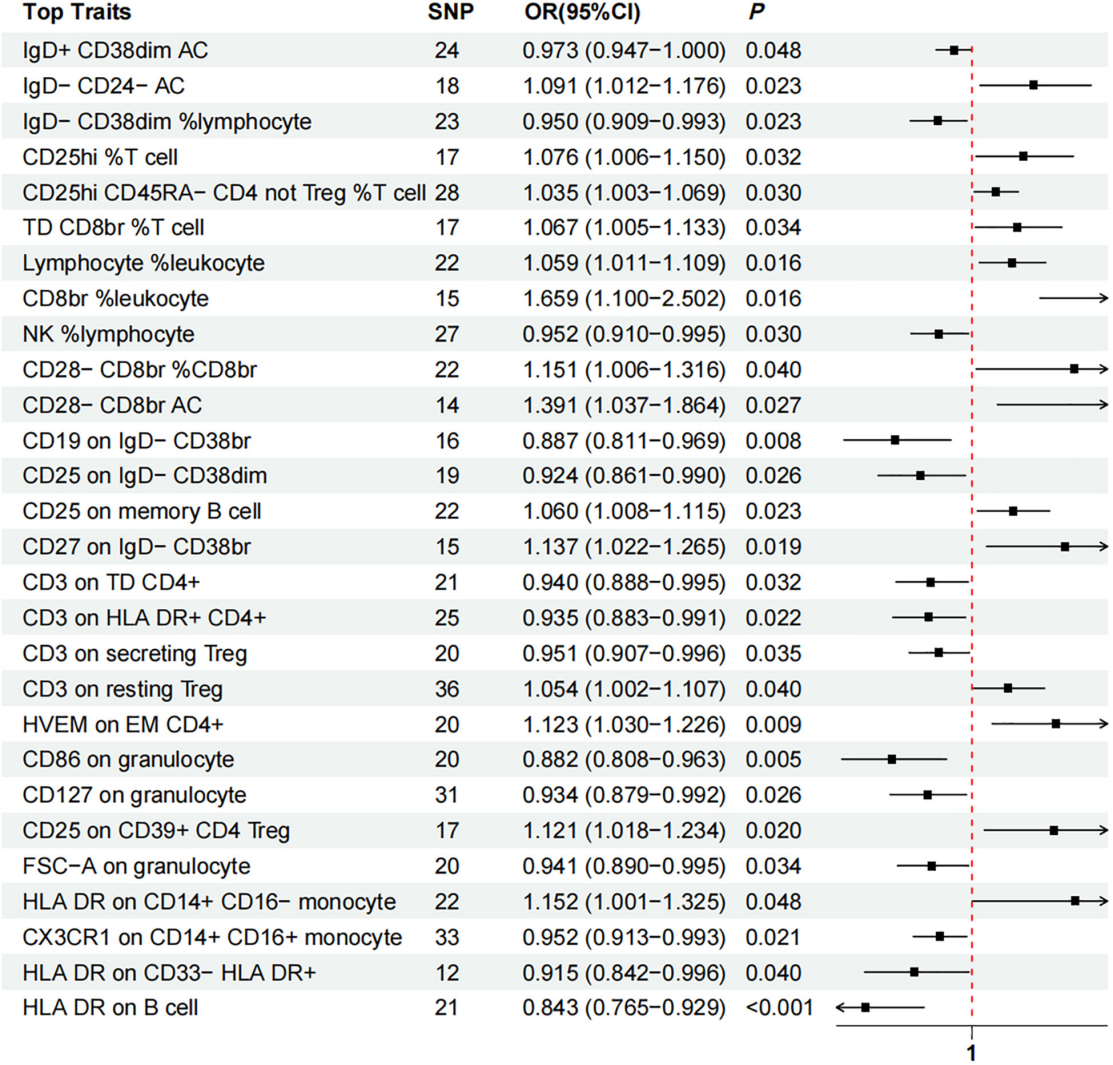
Forest plot of Mendelian randomization analysis showing the effect of immune cells on lichen planus. OR (odds ratio), *P* and 95% CI (Confidence Interval) are the results obtained from the IVW method. SNP: Single nucleotide polymorphism.

Through two-sample MR analysis, we identified 28 immune cells causally associated with LP (**Supplementary Table S2**). While other tests may not be statistically significant, the IVW method indicated that the immune cell types causally associated with LP were primarily involved in T cells, B cells, and NK cells (**Figure 2**). CD8br %leukocyte (OR = 1.659, 95% CI [1.100, 2.502], *P_IVW_* = 0.016), CD28 - CD8br AC (OR = 1.391, 95% CI [1.037, 1.864], *P_IVW_* = 0.027), IgD- CD24- AC (OR = 1.091, 95% CI [1.012, 1.176], *P_IVW_* = 0.023), CD25hi %Tcell (OR = 1.076, 95% CI [1.006, 1.150], *P_IVW_* = 0.032), CD25hi CD45RA- CD4 not Treg %T cell (OR = 1.035, 95% CI [1.003, 1.069], *P_IVW_* = 0.030), CD28- CD8br %CD8br (OR = 1.151, 95% CI [1.006, 1.316], *P_IVW_* = 0.040). Conversely, increasing immune cell types of IgD- CD38dim %lymphocyte (OR = 0.950, 95% CI [0.909, 0.993], *P_IVW_* = 0.023), IgD+ CD38dim AC (OR = 0.973 95% CI [0.947, 1.000], *P _IVW_* = 0.048), NK %lymphocyte (OR = 0.952, 95% CI [0.910, 0.995], *P_IVW_* = 0.030) may reduce the risk of LP disease. After applying the Bonferroni correction to account for multiple testing, none of the observed associations reached the adjusted significance threshold(Q=0.05/731). Cochran’s Q statistic showed no heterogeneity, and the MR-Egger intercept test and MR-PRESSO indicated no horizontal pleiotropy in this MR analysis (**Supplementary Table S3**). In addition, sensitivity analysis further validated the stability of the MR analysis (**Supplementary Table S7**). Conversely, reverse MR analysis showed no causality (*P _IVW_* > 0.05) of LP on 28 cell types (**Supplementary Table S4**).

### Effect of plasma metabolites on lichen planus

**Figure 3:**
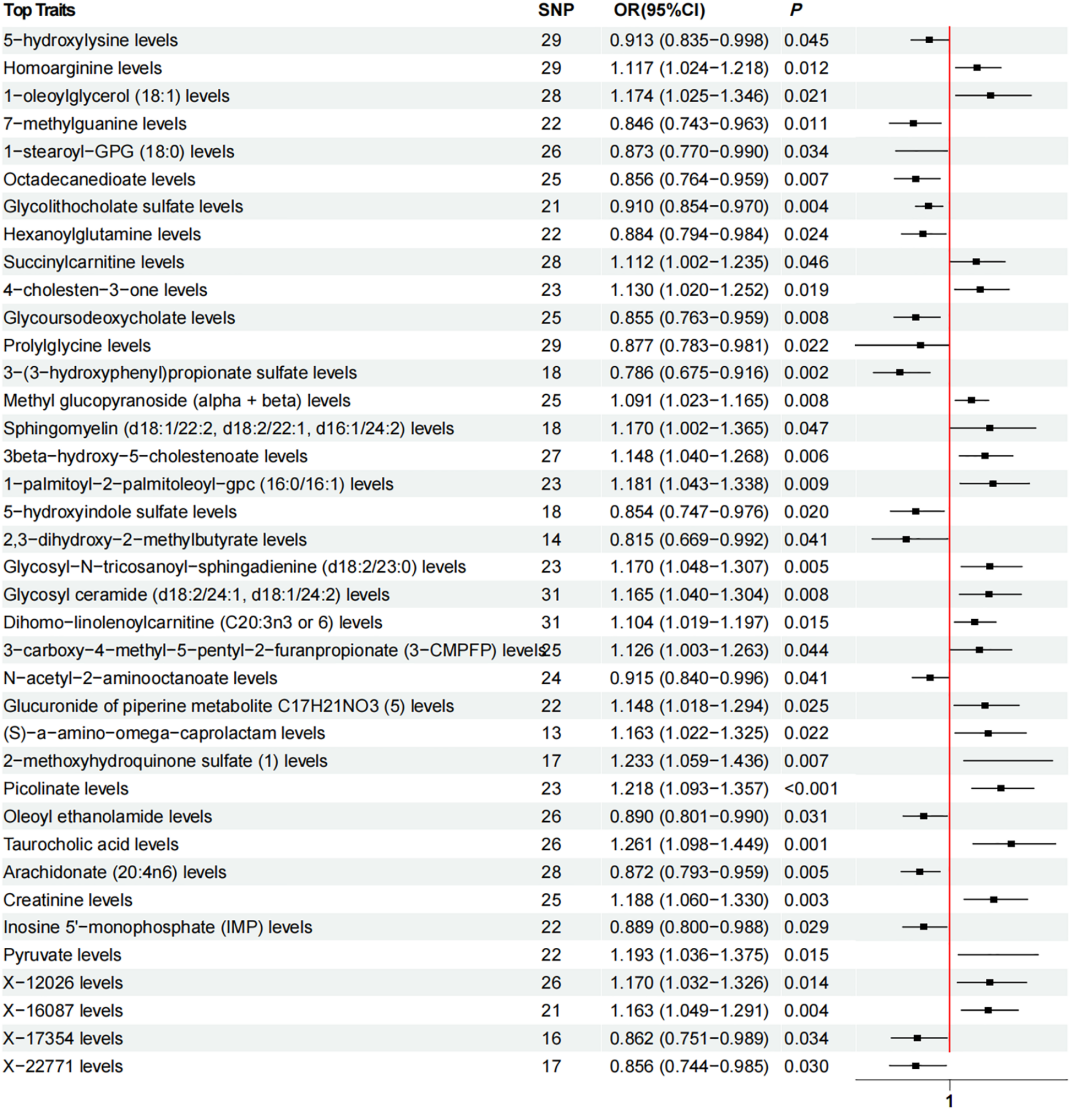
Forest plot of Mendelian randomization analysis showing the effect of plasma metabolites on lichen planus. OR (odds ratio), *P* and 95% CI (Confidence Interval) are the results obtained from the IVW method. SNP, Single nucleotide polymorphism.

Using the IVW method, we identified 38 causal associations between plasma metabolites and lichen planus (*P_IVW_* < 0.05). There were 21 metabolites that had positive causality with LP, while 17 metabolites had negative causality with LP. (**Figure 3**). Increasing plasma metabolite levels of Taurocholic acid levels (OR = 1.261, 95% CI [1.098, 1.449], *P_IVW_* = 0.001), 2- methoxyhydroquinone sulfate (1) levels (OR = 1.233, 95% CI [1.059, 1.436], *P_IVW_* = 0.007), Picolinate levels (OR = 1.218, 95% CI [1.093, 1.357], *P_IVW_* < 0.001) were associated with an increased risk of LP. In contrast, increasing plasma metabolite levels of 3-(3-hydroxyphenyl) propionate sulfate levels (OR = 0.786, 95% CI [0.675, 0.916], *P_IVW_* = 0.002), 2,3-dihydroxy-2-methylbutyrate levels (OR = 0.815, 95% CI [0.669, 0.992], *P_IVW_* = 0.041), and 7-methylguanine levels (OR = 0.846, 95% CI [0.743, 0.963], *P_IVW_* = 0.011) were associated with a reduced risk of developing LP. Subsequently, after applying the Bonferroni correction to account for multiple testing, none of the observed associations reached the adjusted significance threshold(Q=0.05/1091). The MR-Egger intercept test and MR-PRESSO indicated absence of horizontal pleiotropy, Cochran’s Q statistic revealed no heterogeneity (**Supplementary Table S5**), and leave-one-out analyses demonstrated that removal of specific SNP did not alter the causal estimates (**Supplementary Table S7**).

### Effect of NK %lymphocyte on Picolinate levels

Previously, our study identified 28 immune cell signatures and 38 plasma metabolite signatures causally associated with LP. Subsequently, we investigated the causal relationship between 28 immune cell traits and 38 plasma metabolites. MR analysis revealed that Picolinate levels act as a potential mediator in the relationship between immune cells and LP. Both NK% lymphocyte and CD127 on granulocyte may affect the occurrence of lichen planus mediated by Picolinate Levels, but NK% lymphocyte has a greater impact on LP. Specifically, NK %lymphocyte was found to have a significant effect on Picolinate levels (IVW, OR = 0.960, 95% CI [0.932, 0.988], *P* = 0.006), (Weighted median, OR = 0.948, 95% CI [0.912, 0.986], *P* = 0.008), (Weighted mode, OR = 0.956, 95% CI [0.920, 0.994], *P* = 0.030). Cochran’s Q statistic indicated no heterogeneity, and both the MR-Egger intercept test and MR-PRESSO indicated no horizontal pleiotropy in this MR analysis (**Supplementary Table S6**). Leave-one-out analysis shows that deleting specific SNPs does not change causal estimates (**Supplementary Table S7**).

### A reverse MR analysis

We identified causal links between NK %lymphocyte and Picolinate levels, as well as between Picolinate levels and LP. To validate these causal links further, we conducted reverse MR analysis. The results showed that no causal effect of lichen planus on Picolinate levels was detected (IVW, OR = 0.996, 95% CI [0.941, 1.053], *P* = 0.876), and furthermore, no causal effect of Picolinate levels on NK %lymphocyte was found (IVW, OR = 1.028, 95% CI [0.933, 1.134], *P* = 0.576). At the same time, we did not find horizontal pleiotropy and heterogeneity in our analysis.

### Mediator effect of NK %lymphocyte on LP is mediated by Picolinate levels

**Figure 4:**
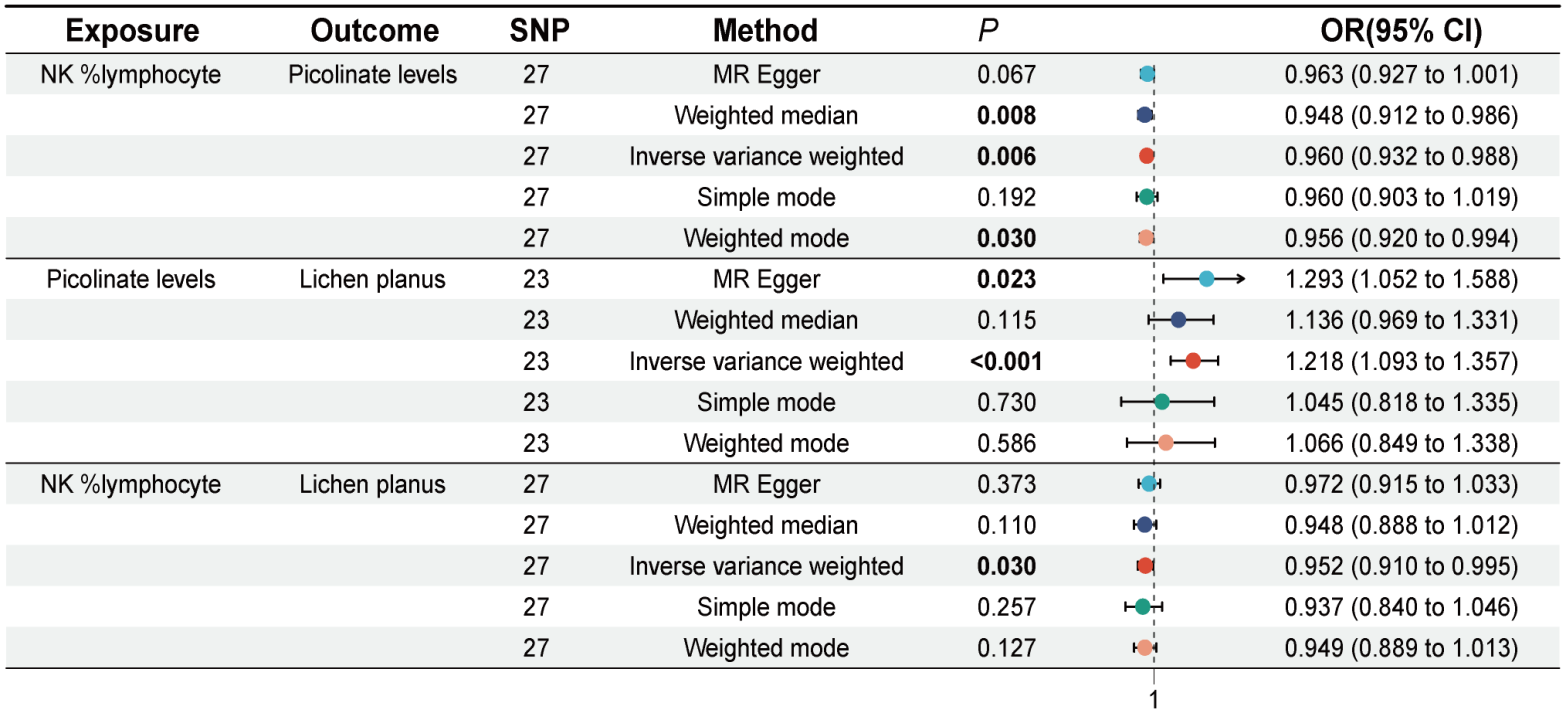
Forest plot of Mendelian randomization analysis showing Causal effect of NK %lymphocyte on Picolinate levels and causal effect of Picolinate levels on lichen planus. SNP, Single nucleotide polymorphism, OR (odds ratio), 95% CI (Confidence Interval).

Mediation analyses assess the pathway from exposure to outcome through potential mediators, identifying effects of exposure on outcome. We examined the mediating effect of Picolinate levels on the relationship between NK %lymphocyte and LP. The mediation analysis revealed that Picolinate levels had a mediator effect of -0.00813, explaining 16.4% of the total effect (95% CI: [28.3%, 4.54%], *P*=0.007) (**Figure 4**).

## Discussion

Previous studies have identified lichen planus as an autoimmune disease closely linked to immune cells. To investigate the causal relationship between immune cells and LP, we conducted a two-sample MR analysis, revealing significant causal associations of 28 immune cell types with LP. Extensive research on immune cells suggests that metabolites play a crucial role in mediating their biological effects (31, 32). To explore if these 28 immune cells influence LP through metabolite changes, we conducted a two-step MR analysis, finding that 21 metabolites were detrimental to LP, while 17 metabolites were protective. We then assessed the causal relationship between 28 immune cells and 38 plasma metabolites. Although these associations were not significant after adjustment, the trends observed are consistent with findings from previous studies and suggest a potential biological relationship. These trends may indicate pathways that warrant further attention. Results indicated a close association between NK %lymphocyte and Picolinate levels. Mediation analysis revealed Picolinate contributed significantly, explaining 16.4% (95% CI: [28.3%, 4.54%], *P*=0.007) of the association between NK %lymphocyte and LP, underscoring Picolinate’s role in linking NK %lymphocyte to LP.

In the past few decades, studies have found that NK cells are associated with the occurrence and development of various diseases (33, 34). In dermatology, Michal et al.’s experimental studies demonstrated NK cells enhance early cutaneous antimicrobial defense in an HIF-1α-dependent manner at multiple levels (35). NK cells have been hypothesized to be involved in the development of lichen planus, especially cutaneous lichen planus (36). NK cells may co-localize with DC cells (37), and can in turn exhibit cytotoxicity through perforin release, modulating DC and T cell activation and releasing cytokines such as TNF-α, IFN-γ and IL-22 (38). Other studies have also shown NK cells express CXCR3, CCR5, CCR6, and ChemR23, chemotactic protein receptors produced by vascular endothelial cells (39). They may provide early stimulatory signals for mobilizing T cells to sites of inflammation (40). Therefore, NK cells may serve as major effector cells in lichen planus. However, to date, no definitive studies have illustrated a causal link between NK lymphocytes and lichen planus. Our reverse MR analysis found no evidence of reverse causality between LP and NK lymphocytes, indicating the association is likely unidirectional.

Picolinate, also called picolinic acid, is an end product of tryptophan oxidative metabolism through the kynurenine pathway (KP) (41). Tryptophan catabolism primarily involves two enzymes: indoleamine 2,3-dioxygenase (IDO), mainly in inflammatory cells, and tryptophan 2,3-dioxygenase (TDO), primarily in the liver (41–43). Pro-inflammatory cytokines like IL-6 and IL-1β are known to influence KP metabolite production by activating specific KP enzymes (44–47). Research on picolinic acid has so far focused on its neuroprotective or immunomodulatory effects and on the fact that it has potent chelator properties (42, 48, 49). Johanna et al. showed that picolinic acid inhibits the proliferation and metabolic activity of CD4 T cells (50). In Bosco et al.’s study, picolinic acid triggered the expression of MIPs chemokines in macrophages, which in turn triggered T-cell aggregation and binding to T-cell products (e.g. IFN-γ) (51). In the context of skin diseases, picolinic acid remains underexplored and has only been reported in acne treatment (52). However, our study identified Picolinate levels as a potential high-risk factor for LP and a key modulator influencing NK %lymphocyte in LP pathogenesis.

In this study, we conducted MR analyses to investigate the causal impact of immune cells on LP and to assess the mediating role of plasma metabolites. In observational settings, this study mimics a randomized controlled trial with lower costs and reduced risk of reverse causation. However, Since the GWAS data of this study are derived from predominantly European populations, they may not fully reflect the genetic structure of other races or populations (53). Differences in genomic diversity and linkage disequilibrium patterns may affect the effectiveness of IV, thereby limiting the applicability of the research results worldwide. Although MR analysis reduces the impact of environmental confounding factors, specific environmental exposures (such as dietary habits, smoking, chronic inflammation, etc.) may change the strength of their association with the disease by regulating the expression or effect of SNP. This potential gene-environment interaction may not have been captured in this study. At the same time, our findings need to be further verified in other clinical trials and animal models. In the TBNK panel, the pathway from NK %lymphocyte to LP was partially mediated by Picolinate levels. However, the mediating effect accounted for only 16.4% of the total effect. Other potential mediators warrant further investigation. Nonetheless, our study evaluated the plasma metabolites-mediated causal impact of immune cells on LP and offered insights into immune cell-mediated LP pathogenesis. This provides a new avenue for exploring future therapeutic options for LP.

## Conclusions

This study estimated the causal relationship between immune cells, plasma metabolites and lichen planus. Picolinate levels were identified as mediators in reducing the risk of lichen planus through NK %lymphocyte. Thus, interventions aimed at modulating Picolinate levels may alleviate the impact of low NK %lymphocyte on lichen planus. This study offers novel evidence and insights into the pathogenesis of lichen planus. It also contributes new insights into LP prevention and treatment.

## Data Availability

The datasets presented in this study can be found in online repositories. The names of the repository/repositories and accession number(s) can be found in the article/**Supplementary Material**.
