# Supplementary figures and images for: Picolinate-mediated immunomodulation: insights from Mendelian randomization on the role of NK cell percentage in the pathogenesis of lichen planus
Source: Front Immunol. 2024 Dec 12;15:1464479. doi: 10.3389/fimmu.2024.1464479 (PMC11669599; doi:10.3389/fimmu.2024.1464479)

A


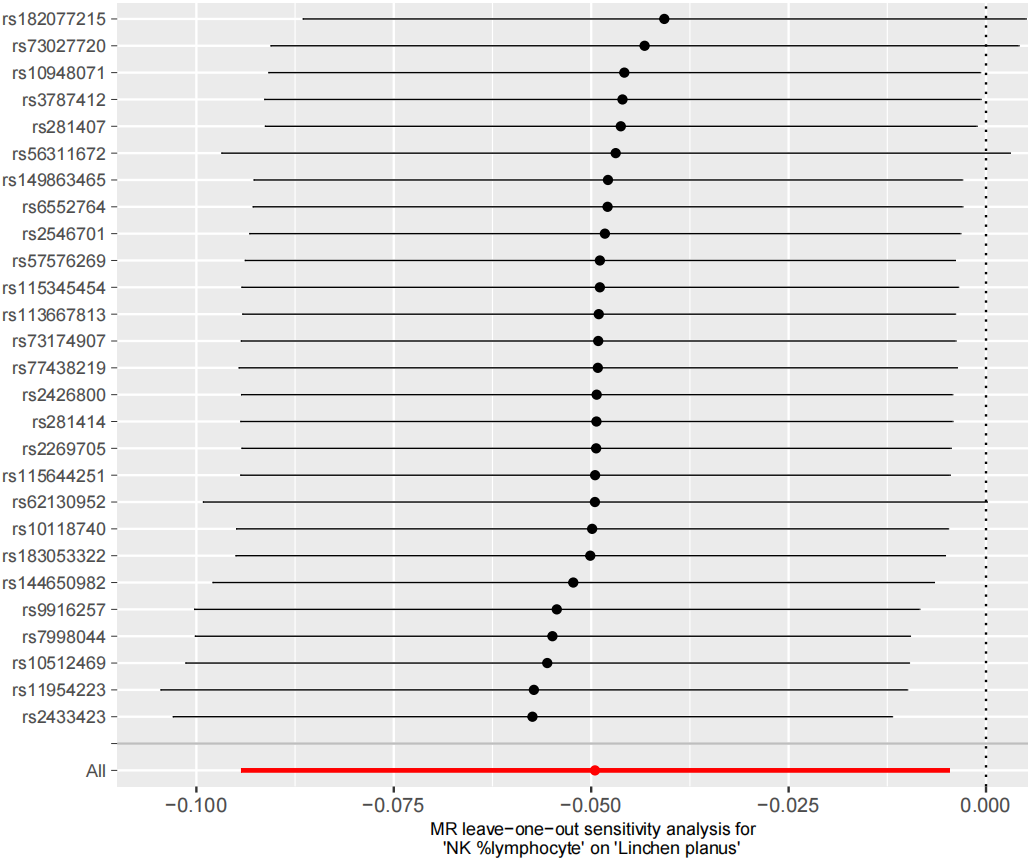


B


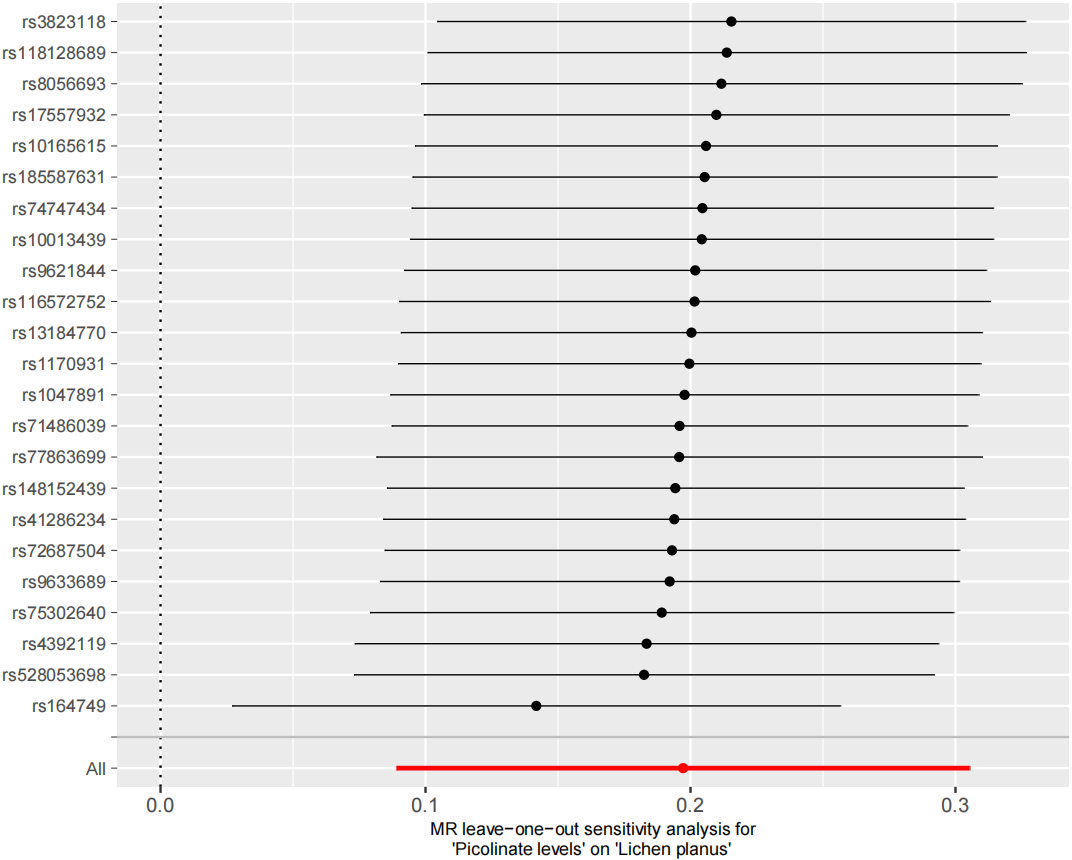


C


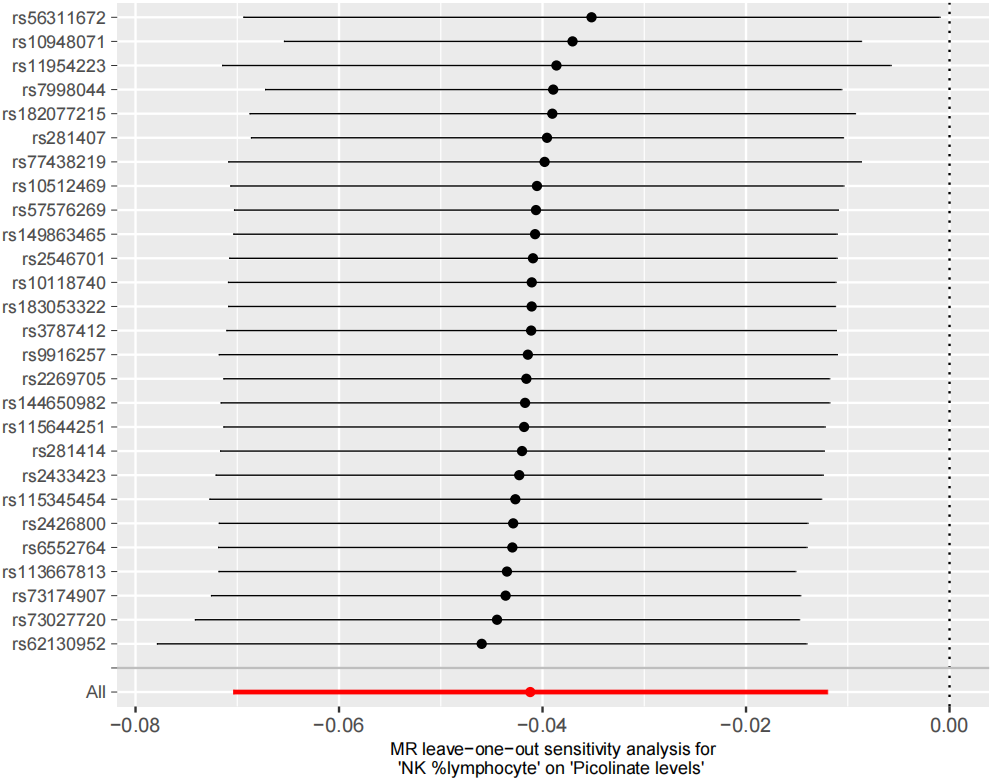

Supplement: Supplementary file 7 [file Table7.docx]
